# Budget impact of antimicrobial wound dressings in the treatment of venous leg ulcers in the German outpatient care sector: a budget impact analysis

**DOI:** 10.1080/20016689.2018.1527654

**Published:** 2018-11-01

**Authors:** Maria Gueltzow, Poroshat Khalilpour, Katharina Kolbe, York Zoellner

**Affiliations:** a Global Regulatory Affairs, BSN medical GmbH, Hamburg, Germany; b Econ-Epi, Hamburg, Germany

**Keywords:** Budget impact, cost-of-illness, Health economics, outpatient care, venous leg ulcer, chronic wounds, antimicrobial wound dressings, Cutimed Sorbact, DACC, silver dressings

## Abstract

**Background:** Hard-to-heal wounds are associated with high treatment costs and, in Germany, are mostly treated in the outpatient care sector. Wound dressings are the main cost-drivers in venous leg ulcer (VLU) care which prescription is budget-restricted.

**Objective:** To determine to what extent the choice of antimicrobial dressing affects the spending in outpatient care by investigating the budget impact of the bioburden-reducing dressing Cutimed Sorbact.

**Methods:** The budget impact analysis was performed comparing three different scenarios of the intervention mix of antimicrobial dressings. A Markov model was used to estimate the VLU progression during one year. The budget impact was determined by comparing the dressing and medicine resource use and costs of the three scenarios.

**Results:** This analysis confirms the high treatment costs of VLU care. Scenario_A_ leads to a decreased resource use of antimicrobial dressings and results in 20.86% lower treatment costs after 12 months. The increased use of Cutimed Sorbact has a positive budget impact.

**Conclusion:** This analysis indicates that the treatment of VLU patients may result in an exceedance of the budget per patient that is available to the treating practitioner. The choice of wound dressing, however, may positively affect the prescribers’ budget spending in outpatient care.

## Introduction

Due to the aging population, the prevalence and incidence of hard-to-heal wounds is predicted to increase. Such wounds impact the quality of life of affected patients and their families, impose an increased burden on their daily lives and mean significant costs to the society. It is therefore important to focus on high quality therapy standards to ensure best practice [1,2].

Venous leg ulcers (VLUs) have the highest prevalence of hard-to-heal wounds [3]. About 0.6–1% of the German population are diagnosed with a VLU, with the prevalence in people above the age of 60 being higher (3.9%) [4]. Only about half of VLUs heal within 4 months, and healed ulcers show high recurrence rates of 30 to 57% within the first year after wound closure [5].

Standard of Care (SoC) for the treatment of VLUs is compression therapy [5]. In combination with the latter, local wound treatment plays an essential role in supporting the wound healing process [3]. Dressings that create a moist wound environment and provide an adequate exudate management are recommended by German and international guidelines [3,5].

In the treatment of chronic ulcers with heavy bacterial contamination, wound dressings that contain antibacterial agents (e.g. silver) are commonly used [5]. The effectiveness of these dressings in wound healing and treating infection was investigated in multiple studies, but the evidence is described as limited, due to inadequate or low-quality study designs [cf.4]. However, studies have demonstrated reduced signs of infection and bacterial contamination when using antimicrobial dressings, which is why these dressings are established in daily practice of practitioners to fight wound infection [6–9].

Studies indicate that the cost-of-care for chronic wounds is high. In Germany, the mean annual cost per VLU patient is expected to be 8,288 to 9,569 €, with about half of the costs arising in the outpatient care sector [10,11]. Hospitalisation and non-drug treatment costs were identified as the main cost drivers, whereas the latter include wound dressings and surgical hoses and other non-drug therapeutics [10,11]. The use of antimicrobial dressings incurs higher costs compared to moist wound care, although it may lead to an overall reduction in costs in the longer term [1].

The aim of this analysis was to determine to what extent the choice of antimicrobial dressing affects the budget for medicines and dressings in outpatient care by investigating the practitioners’ budget impact of the bio-burden reducing wound dressing Cutimed Sorbact (BSN medical GmbH, Hamburg, Germany) which is coated with Dialkylcarbamoyl chloride (DACC). Due to its highly hydrophobic characteristics, microorganism with hydrophobic extracellular surfaces that are commonly found in chronic wounds attach to the DACC coating on the wound dressing and can therefore be removed with dressing change. The results of this analysis provide information for practitioners and can support them in the decision-making process in VLU treatment [12].

## Methods

### Study design

The budget impact (BI) of Cutimed Sorbact (BSN medical GmbH, Hamburg, Germany) was calculated using a disease-specific cohort in combination with a Markov model. To identify the number of eligible patients, predefined inclusion criteria were applied. Three different scenarios (S) were created to estimate the BI of different market shares of Cutimed Sorbact (BSN medical GmbH, Hamburg, Germany) in the current intervention mix (S_Base_), an increased use of the wound dressing in 50% of the patients (S_A_), and no use of the dressing with a market share of zero (S_B_). The overall costs for medicines and dressing were derived for each scenario and then compared to identify the net budget impact of Cutimed Sorbact (BSN medical GmbH, Hamburg, Germany). The structural framework of the budget impact analysis can be found in a flow model Appendix 1.

### Perspective

The analysis was conducted from the perspective of the budget for medicines and dressings of SHI-accredited practitioners.^1^ 1In the outpatient care sector, wound dressings that are defined as ‘Verbandmittel’ according to the German Federal Joint Committee (‘Gemeinsamer Bundesausschuss’) [33] are covered by the statutory health insurance (SHI). Since the budget per patient is fixed, the decision on which dressing to use for the treatment of a VLU is decided by the SHI-accredited practitioner according to the wound status (e.g. exudate level), the efficiency and medical necessity of the wound dressing [5,24]. This analysis focusses on GPs and dermatologists practicing in the outpatient care sector, since the majority of patients with chronic wounds are treated in this setting [10]. Due to the different budget sizes between regions and specialities, the average budget has been calculated for both general practitioners and dermatologists [cf.13–15]. The average quarterly budget per patient (above 60 years) is 120.41€ for GPs and 41.38€ for dermatologists.

### Time horizon

The time horizon of the budget impact analysis has been set to twelve months. The practitioners’ budgets for medicines and dressings in outpatient care are quarterly budgets; therefore, the results of this analysis will be presented per quarter-year.

### Base-case population

The VLU prevalence is highest in patients aged 60 years and older [5]. The base-case population therefore includes patients above the age of 60 with a VLU.

**Figure 1.**
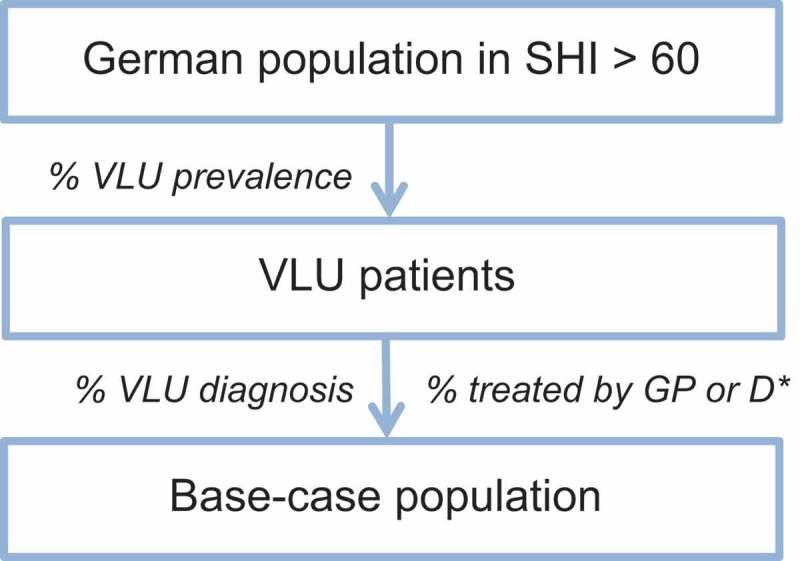
Identification of the base-case population. *D: Dermatologist

The procedure of identifying the eligible population is shown in Figure 1. First, the prevalence and incidence rates of VLUs are applied to the SHI insured German population aged 60 and older [3,4,13,14]. Subsequently, the percentage of patients with VLU diagnosis [15] that receive treatment through a GP and dermatologist [10] is applied to the German SHI-population. The assumption was made that 100% of the patients have been diagnosed.

### Decision-analytic model

The endpoints of the model are the eradication of wound infection and healing (complete wound closure). A review of the available literature over the last 10 years (2007–2017) has been conducted. Inclusion criteria were the following:

- Study design: Meta-analysis, systematic review, RCT, observational study, health economic evaluation (based on decision-analytic model or RCT)
- Intervention: wound dressing (transition probabilities: Dialkylcarbamoylchlorid (DACC), Silver, Polyhexanide (PHMB))
- Outcomes of interest: wound healing and/or wound infection

Publications with inconsistent methods [16,17], case studies and literature that did not meet the inclusion criteria were excluded.

The databases of the National Institute for Health Research (NIHR), National Institute for Health and Care Excellence (NICE), the Cost-Effectiveness Analysis Registry (CEA) and Pubmed database were used to conduct the literature search. The health economic evaluation by Nherera, Woodmansey, Trueman et al. based on a Markov model was used as orientation to develop a Markov model [cf. 23]. Three studies which investigated the effectiveness of a DACC, silver and a PHMB dressing met the inclusion criteria [cf.7–9].

**Figure 2.**
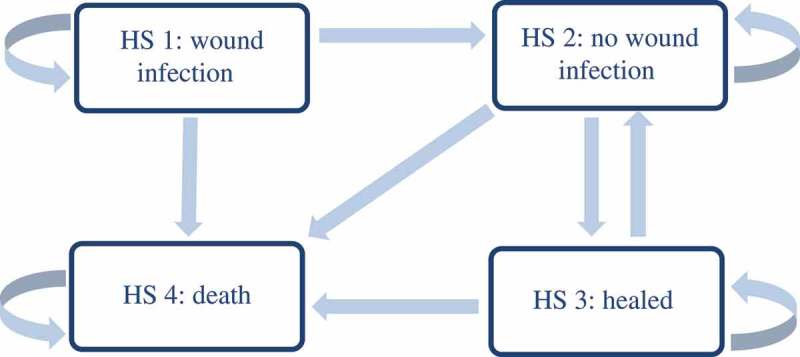
Markov Model with transition probabilities. Monthly cycles. D: DACC Dressing [7], Si: Silver Dressing [8], P: PHMB Dressing [9].

The retrieved literature was used to build and feed a Markov model in Microsoft Excel. The model compromises four different health states (HS) that have been simulated over the course of 12 months in monthly cycles (Figure 2):

It is assumed that 84% of VLU patients enter the model in HS 1 (wound infection) and 16% in HS 2 (no wound infection) [7]. Antimicrobial dressings are used in patients in HS 1 to treat the wound infection. It is assumed that the transition probabilities for infection healing do not differ between dressings or manufacturers [cf. 8–9]. In VLUs without a wound infection (HS 2), moist wound dressings (SoC) are applied [cf.4]. The number of weekly dressing changes is set to 2.5 in both HS 1 and 2 [7,9,18].

Patients with a healed ulcer (HS 3) do not receive any treatment, and patients in HS 4 are taken out of analysis. It is assumed that the choice of dressing does not have an impact on ulcer recurrence. The chosen recurrence rate refers to antimicrobial and standard dressings [18].^2^ 2Further assumptions are presented in Appendix 2. The transition probabilities can be found in Table 1:

**Table 1.** Transition probabilities for different wound dressings.

	Cutimed Sorbact	Silver Dressings	PHMB Dressings
Transition Probabilites			
HS 1 → HS 2	0.81 [7]	0.16 [8]	0.57 [9]
HS 2 → HS 3	0.21 [7]	0.16 [8, cf.9]	0.16 [8, cf.9]
HS 3 → HS 2 (relapse)	0.01 [32]	0.01 [32]	0.01 [32]
HS 1/2/3 → HS 4 (mortality from natural causes)	0.01 [14]	0.01 [14]	0.01 [14]

### Intervention mix

The mix of dressings indicated for the treatment of infected wounds include DACC and silver and PHMB wound dressings. The dressings with the highest sales volume in Germany (2017) were chosen as the base-case scenario [19]. The following dressings are included:

DACC dressing:

- Cutimed Sorbact Tamponade (BSN medical GmbH, Hamburg, Germany)

Silver dressings:

- Allevyn Ag Gentle Border (Smith & Nephew GmbH)
- Aquacel Ag Extra (Convatec Germany GmbH)
- Biatain Silicon Ag (Coloplast GmbH)
- Mepilex Ag (Moelnlycke Health Care)
- Urgotuel Silver (Urgo GmbH)

PHMB dressings:

- DracoFoam Infekt foam dressing (Dr. Ausbüttel & Co. GmbH)
- Suprasorb P+ PMBH (Lohmann & Rauscher GmbH)

The respective market shares were calculated using the sales volume of the specific dressing, assuming that the above-mentioned products equal 100% of antimicrobial wound dressings used in VLUs. Based on the market share of the dressings, the distribution of patients on different dressings was estimated. Using the market shares of the base-case scenario, the shares for Cutimed Sorbact (BSN medical GmbH, Hamburg, Germany) were altered to 50% in S_A_ and 0% in S_B_ as presented in Table 2.

**Table 2.** Market share and N treated patients per wound dressing in each scenario.

	S_Base_	S_A_	S_B_
	% (N)	% (N)	% (N)
Cutimed Sorbact	**19.87 (59,101)**	**50.00 (148,753)**	**0 (0)**
Allevyn Ag	7.92 (23,564)	4.94 (14,703)	9.88 (29,406)
Aquacel Ag	11.29 (33,585)	7.04 (20,955)	14.09 (41,910)
Biatain Ag	5.93 (17,653)	3.70 (11,015)	7.4 (22,029)
Mepilex Ag	11.51 (34,250)	7.18 (21,370)	14.37 (42,741)
Urgotuel Silver	18.67 (55,543)	11.65 (34,656)	23.30 (69,312)
DracoFoam Infekt	16.78 (49,919)	10.47 (31,147)	20.94 (62,294)
Suprasorb P+ PMBH	8.03 (23,892)	5.01 (14,907)	10.02 (29,815)
Total	100 (297,507)		

The cost in the three different scenarios include the costs for pharmaceuticals and wound dressings. The dressing costs are calculated by multiplying the number of patients treated with each dressing by the number of monthly dressing changes (n = 2.5*4). Prices are adjusted to the cost of one dressing of 10 cm x 10 cm to ensure the comparability of dressing costs. Additionally, prices for average resource of secondary dressings (practice supplies) that are used for VLU treatment according to two wound care experts were derived from the Lauer-Taxe [20]. The dressing acquisition costs can be found in Table 3.

**Table 3.** Aquisition costs [19] *price adjusted to measurement.

product	manufacturer	type	price (10 x 10 cm)
**First Dressing**			
Allevyn Ag	Smith& Nephew	foam dressing	14.05 €*
Urgotuel Silver	Urgo	gauze	5.92 €*
Cutimed Sorbact	BSN Medical	tamponade	9.14 €
Biatain Silic Ag	Coloplast	silicone dressing	12.93 €*
Mepilex Ag	Mölnlycke	foam dressing	13.49 €
Aquacel Ag Extra	Convatec	compress	13.72 €
Suprasorb P+ Phmb	Lohmann & Rauscher	foam dressing	12.28 €
Dracofoam Infekt	Dr. Ausbüttel & Co.	foam dressing	9.77 €
wound dressing according to SoC		moist gauze	1.73 € [10]
**Secondary Dressing**			
		gauze bandage	0.65 €*
		compresses	0.11 €*
		adhesive tape	9.55 €*
		Total	10.30 €*

Subsequently, the annual median drug costs per patient according to Augustin, Brocatti, Rustenbach et al. [10] are used to calculate the monthly drug expenses for the treated patients. It is assumed that the drug resource use is the same for patients with healed and non-healed VLU. To estimate the total costs per scenario, expenses per patient in the different health states were summated.

The net costs for S_A_ compared to the base-case scenario (S_A_ – S_Base_) and S_B_ compared to the base-case scenario (S_B_ – S_Base_) were calculated and broken down to one quarter. The cost estimates for each scenario represent the budget impact of Cutimed Sorbact (BSN medical GmbH, Hamburg, Germany).

### Uncertainty analysis

A deterministic one-way sensitivity analysis was performed to estimate which parameters drive the uncertainty of the results. To ensure a plausible range of the different parameters, the opinion of two experienced practitioners was taken into account [cf.28].

## Results

### Eligible population

As described in Figure 1, the base-case population was calculated by applying the inclusion criteria to the German population. As a result, 297,507 eligible patients could be identified.

### Changes in health outcome

**Figure 3.**
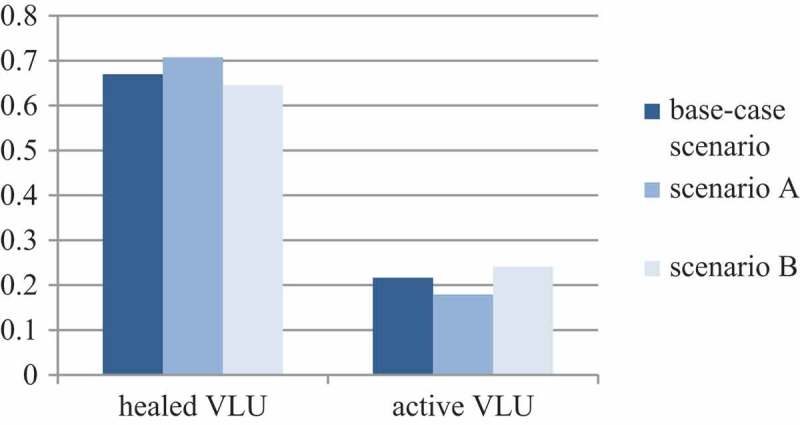
Percentage of healed and active (not healed) VLU per scenario after 12 months.

In the current intervention mix, 66.9% of the treated VLUs heal within 12 months, while 21.6% of the patients are still being treated by a GP or dermatologist due to their VLUs (active VLU without infection: 14.8; active VLU with infection: 3.1%) and 11.4% of the patients die due to natural causes. In S_A_, 71% of the VLUs heal within a year while 18% of the patients still receive treatment due to their unhealed VLU (active VLU without infection: 17.9; active VLU with infection: 6.2%). In S_B_, 65% of VLU heal within one year while 17.9% retain an active VLU without infection and 6.2% retain an active VLU with infection. In contrast to S_Base_, S_B_ results in a higher number of patients (+ 12%) requiring VLU treatment after 12 months (Figure 3).

These results show that the increased usage of Cutimed Sorbact (BSN medical GmbH, Hamburg, Germany) in outpatient care reduces the number of patients who need to receive treatment with pharmaceuticals or bandages and dressings due to their VLU.

### Resource use

**Figure 4.**
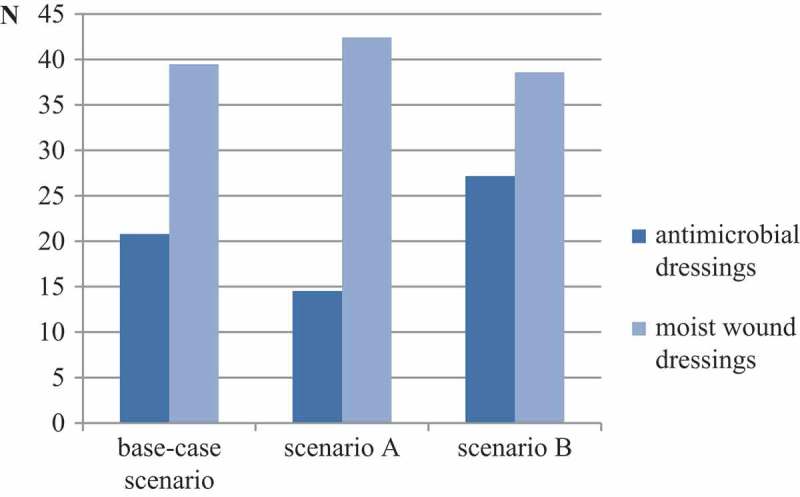
Annual dressing resource use per patient.

In the base-case scenario, the annual resource use per patient is 21 antimicrobial dressings and 39 moist dressings. If a practitioner, however, decides to use Cutimed Sorbact (BSN medical GmbH, Hamburg, Germany) in every second patient (S_A_), the amount of antimicrobial dressings used can be reduced by around 30%. In S_B_, a higher number of antimicrobial and moist dressing per patient is needed. Figure 4 shows a comparison of the dressing resource use in each scenario.

### Costs

Table 4 shows the total costs per patient in each scenario for dressing and drug expenses separately. In S_Base_, the average costs per VLU patient ranges from 361.63€ in the first quarter to 98€ in Q4, whereas S_A_ leads to lower treatment costs from the first quarter onwards. The opposite becomes apparent for S_B_.

**Table 4.** Cost per patient per quarter (Q) for each scenario.

	Q1	Q2	Q3	Q4
**Base-case scenario**				
Dressing Cost	160.36 €	91.19 €	56.02 €	34.94 €
Drug Cost	201.28 €	140.16 €	94.42 €	63.89 €
Total	361.63 €	231.34 €	150.44 €	98.83 €
**Scenario A**				
Dressing Cost	122.21 €	66.16 €	40.17 €	24.96 €
Drug Cost	195.90 €	126.26 €	80.83 €	53.17 €
Total	318.11 €	192.43 €	121.01 €	78.13 €
**Scenario B**				
Dressing Cost	185.50 €	107.68 €	66.47 €	41.51 €
Drug Cost	204.82 €	149.32 €	103.37 €	70.96 €
Total	390.32 €	257.00 €	169.84 €	112.47 €

In the base-case scenario, the average treatment cost per patient per quarter is 210.49 €.

### Budget impact

In order to determine the budget impact of Cutimed Sorbact (BSN medical GmbH, Hamburg, Germany), the net costs of S_A_ and S_B_ compared with S_Base_ were calculated. The results are presented in Table 5 below which demonstrates the net savings/losses in dressing and drug expenses:

**Table 5.** Budget impact of Cutimed Sorbact per scenario.

		Q 1	Q2	Q3	Q4
Net Costs per Quarter (Q) in EUR					
S_A_-S_Base_	Total	−12,689,226.13	−11,016,398.92	−8,086,219.86	−5,517,690.32
	Per Patient	−43.53	−38.92	−29.43	−20.70
S_B_-S_Base_	Total	+ 8,364,912.55	+ 7,262,150.99	+ 5,330,517.63	+ 3,637,290.96
	Per Patient	+ 28.69	+ 25.65	+ 19.40	+ 13.64

S_A_ compared to the current intervention mix generates cost-savings in both dressing and drug expenses. In S_B_, in which Cutimed Sorbact (BSN medical GmbH, Hamburg, Germany) is not used, additional costs in both categories arise.

**Figure 5.**
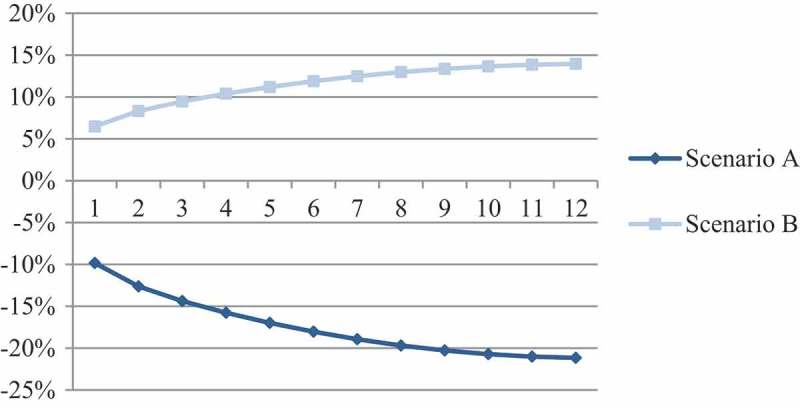
Relative budget impact per scenario (compared with base-case scenario).

S_A_ results in a decrease in overall treatment costs of 21 percent within one year compared to the base-case scenario, whereas S_B_ results in increased expenses of 14 percent (Figure 5). The increased usage of Cutimed Sorbact (BSN medical GmbH, Hamburg, Germany) in the treatment of infected VLUs therefore impacts the practitioners’ budget positively and generates budget-savings over the course of 12 months.

### Uncertainty analysis

**Figure 6.**
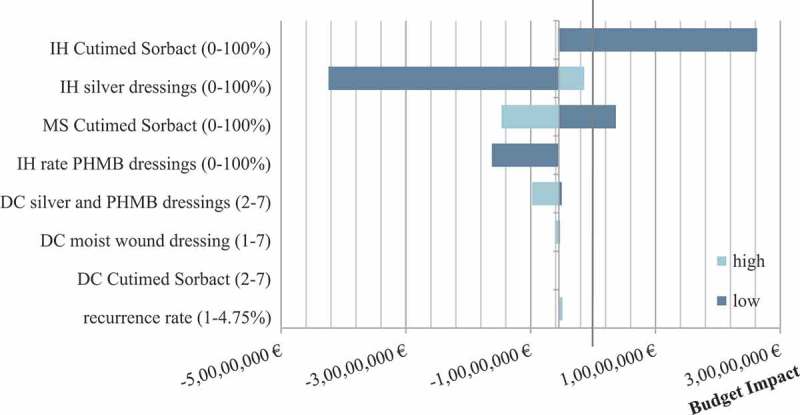
Univariate sensitivity analysis: Tornado chart. Baseline budget impact (SA-SBase): −6,345,272.61€. IH: infection healing, MS: market share, DC: number of weekly dressing changes.

A univariate sensitivity analysis has been performed using the budget impact of S_A_- S_Base_ in Q4 (months 10,11,12) as the baseline. The results are presented in Figure 6. We used a Tornado diagramme to display the effects of increasing or decreasing a certain parameter (in all scenarios), e.g. dressing change, on the baseline budget impact. A baseline budget impact of zero or higher results in additional costs while a budget impact below zero demonstrates budget savings (compared to S_Base_).

The Figure shows that the budget impact is primarily sensitive to the infection healing rate of Cutimed Sorbact (BSN medical GmbH, Hamburg, Germany) and silver dressings. If the effectiveness of Cutimed Sorbact (BSN medical GmbH, Hamburg, Germany) in curing infection in all patients would be below 20%, S_A_ would no longer result in cost-savings (compared to S_Base_). The increase in the infection healing rate of silver dressings to 100% does not lead to additional costs in the baseline budget impact, meaning that S_A_ is still less costly than S_Base_. An increased market share of Cutimed Sorbact shows an increase in cost-savings whereas a decrease results in decreased cost-saving. A decrease in the PHMB infection healing rate does show sensitivity in favour of the baseline budget impact whereas an increase shows no effect. The same applies for the number of weekly dressings changes in silver and PHMB dressing.

Compared to the average quarterly budget for medicines and dressings per patient (GP:120.41 €, Dermatologist: 41.38 € [21–23]), the costs notably exceed the outpatient care budget. The use of Cutimed Sorbact (BSN medical GmbH, Hamburg, Germany) in 50% of the patients decreases the overspending by 28 percent. The current intervention mix is not cost-covering for practitioners in outpatient care, but budget pressure can be relieved by making appropriate product choices.

## Discussion

In Germany, practitioners in outpatient care have a fixed quarterly budget per patient to prescribe medicines and dressings, and therefore need to choose wound dressings according to their efficiency and medical necessity [24]. Chronic wounds such as VLUs, may notably exceed the practitioner’s budget due to high treatment costs, with wound dressings being the main cost-drivers in outpatient care [10,11]. Thus, it is crucial to investigate to what extent the choice of wound dressing affects the practitioners’ budget.

In this analysis, the budget impact of antimicrobial dressings in the treatment of locally infected VLUs has been investigated using a Markov model. The dressing Cutimed Sorbact (BSN medical GmbH, Hamburg, Germany) was used to determine the BI when increasing or decreasing its usage compared to the current treatment situation. The study suggests that the treatment of VLUs imposes a high financial burden on practitioners in outpatient care and that the choice of antimicrobial dressing for treating wound infections affects the budget expenses of the treating practitioner. Dermatologists hereby face a higher financial burden than GPs due to the lower budget per patient.

Despite the low availability of studies investigating the BI of wound dressings on VLU treatment, one French budget impact analysis could be identified that is comparable to this analysis. Yan, Colin, Courdray-Omnés et al. [25] investigated two wound dressings in VLU exudate management in the French Social Security setting using a scenario analysis [25]. The methods are comparable with this German analysis regarding the calculation of target population and dressing resource use. The authors determined the treatment cost per patient to be notably lower than the results of this analysis. This difference in costs can be explained by the fact that the exudate management phase in the analysis by Yan, Colin, Courdray-Omnés et al. [25] was limited to 28 days which is why the authors stress that chronic VLU care may result in notably higher treatment costs. This can be confirmed by this analysis which focussed on chronic, locally infected VLUs.

The results of this analysis were further compared to a Markov model and German cost-of-illness studies to assess external validity. Nherera, Woodmansey, Trueman et al. [26] performed a cost-effectiveness analysis based on a Markov model for VLUs that includes the health state ‘wound infection’. Although the investigated wound dressings differ considerably, the annual SoC healing rate allows for comparison. According to the authors, 54% of VLUs are expected to heal within 12 months [26]. In the conducted analysis, the annual healing rate is 65.9% which would overestimate the healing rates for a SoC wound dressing by 10% compared to Nherera et al. [26]. The same tendency becomes apparent when comparing the results of the performed analysis with a cohort study by Guest et al. [27]. The latter indicates that the BI analysis assumes a biased healing rate, which in return may lead to an underestimation of treatment costs and SoC dressing resource use. Therefore, the assumption of a monthly healing rate may not be accurate. Instead of assuming a linear progression in the healing rate when converting the annual healing rate to a monthly one, an exponential approach should be selected [cf.32].

To estimate the validity of treatment costs in the German setting, the study results were compared to a German cost-of-illness study. Purwins, Herberger, Debus et al. [11] present the cost-of-illness from a SHI perspective with a detailed presentation of treatment expenses that allows for comparison. According to the authors, average treatment costs that arise from medicines and dressings result in treatment costs of 1,544.20 € per patient per year. In comparison, the results of our analysis underestimate the dressing costs.

This indicates the possibility of bias due to the number of weekly dressing changes, which may be lower than in day-to-day practice. In our analysis it was assumed that wound dressings are changed 2.5 times a week [cf.7,9,24,32]. If the number of weekly dressing changes is increased to seven, the treatment costs result in similar expenses as reported by Purwins, Herberger, Debus et al. [11]. This evaluation may therefore underestimate the financial burden on the practitioners’ budget that arises from treating chronic wounds, due to an overestimation of the wound healing rate and an understated number of weekly dressing changes.

This analysis was conducted using a decision-analytic model which is based on different parameters and structural assumptions that may distort the result and therefore act as limitations of the study [28]. The univariate sensitivity analysis, that investigated the sensitivity of certain parameters, revealed a high level of uncertainty regarding the effectiveness of infection healing of Cutimed Sorbact (BSN medical GmbH, Hamburg, Germany) and silver dressings. The chosen effectiveness rates therefore must be supported by publications of good quality; unfortunately, availability of evidence is limited to observational studies. However, choosing data from the latter may deliver data of higher generalisability due to a non-controlled study setting and, therefore, demonstrate real-life effectiveness of such interventions [29]. Additionally, the sensitivity analysis was only performed one-way which may underestimate the overall uncertainty of the results [30]. Further, assumptions regarding the choice of wound dressing were made. Since there is no adequate recommendation on which types of dressings to manage VLUs with [cf.3–4], it was assumed that the dressings with the highest market shares are used most commonly in VLU treatment.

The results of this analysis show that the choice of antimicrobial dressings can have a relevant impact on the budget for medicines and dressings when treating infected VLUs. As shown, an increased use of the bioburden-reducing dressing Cutimed Sorbact (BSN medical GmbH, Hamburg, Germany) can reduce costs in both drug and dressing expenses, with the impact increasing over the course of 12 months. The use of Cutimed Sorbact (BSN medical GmbH, Hamburg, Germany) in 50 percent of target patients leads to a higher number of healed ulcers and ulcers without wound infection within a year, which thereby lowers the overall cost per patient. This may lead to substantial budget savings within twelve months. However, the results are sensitive towards the effectiveness of Cutimed Sorbact (BSN medical GmbH, Hamburg, Germany) which generates a definite uncertainty concerning the results of this analysis.

This analysis further suggests that the treatment of infected chronic wounds like VLUs may exceed the budget of outpatient practitioners. This implies, that practitioners who treat a high number of VLUs cannot work on a cost-covering basis, even if they choose the most efficient wound dressings. They will need to cross subsidise such excess costs through savings in other patients. This bears the risk of setting wrong incentives. Additional research of high quality is needed to explore the need for policy adjustments in order to reduce the financial burden of chronic wounds.

## Appendix 2. Assumptions

**Table:**

	Assumption	Rational
eligible patients	the average patient is above 60	patients with leg ulcers are most commonly over the age of 60 [5]
	the proportion of treated venous leg ulcers complies with the proportion according to ICD L97 Ulcus cruris	most leg ulcers are of venous aetiology [3]
Markov model	patients with a healed VLU remain eligible	high recurrence rate [5,10]
	all silver wound dressings have the same efficacy	investigation of different silver wound dressings in VULCAN trial without differentiated outcome presentation [21]
		no significant differences in wound closure rate of Aquacel Ag, Acticoat products, Contreet Foam and Urgotuel SSD [8]
	treatment with antimicrobial dressings until wound infection is eradicated	recommendation for silver wound dressings [21,27]
		Cutimed Sorbact may also be used on non-infected wounds but is indicated for the treatment of infected wounds [31]
	number of eligible patients only changes due to mortality	Patients do not enter the model within 12 months (no application of incidence rates)
	all health states have the same mortality rate	no mortality in relation to the usage of wound dressings has been reported
	only VLUs in HS 2 may heal within one model cycle (4 weeks)	An infected wound does not heal without curing the wound infection [expert opinion].
	annual recurrence rate/12 complies with monthly recurrence rate	No monthly recurrence data could be identified
	infection does not recur after successful treatment	Wound may recur within 4 weeks but not be infected immediately after wound opening [expert opinion].
	Adverse events are excluded from the model	The cost for an non-serious adverse event will not differ between the type of dressing and health state 1 (‘wound infection’) [expert opinion]
		No serious adverse events were identified for the different dressing types that occurred due to the use of the dressing [6–9]
cost calculation and resource use	all patients receive the same drug treatment	available publications do not differentiate between patients with and without wound infection
	dressing changes are performed 2.5 times per week	average number of weekly dressing changes according to [7,9,32]
	wound dressings are adjusted to the price for 10 × 10 cm^2^	ensure comparability of prices for wound dressing
		median size of leg ulcers in Germany: 9,1 cm^2^ and medium wound size is treated with 10 × 10 cm wound dressing [cf. 10]
	healed patients do not result in costs for the treating doctor	The patient does not receive any treatment related to the ulcer
	off-label use is excluded from analysis	It is assumed that practitioners act according to the guidelines, wound dressing indication and instruction for use.
	no consideration of catch-up effects	It is assumed that the market shares of the investigated dressings will not change within the timeframe of this analysis.
